# ADAR1 and PACT contribute to efficient translation of transcripts containing HIV-1 trans-activating response (TAR) element

**DOI:** 10.1042/BCJ20160964

**Published:** 2017-03-23

**Authors:** Evelyn Chukwurah, Indhira Handy, Rekha C. Patel

**Affiliations:** Department of Biological Sciences, University of South Carolina, Columbia, SC 29208, U.S.A.

**Keywords:** ADAR1, HIV-1, interferons, PACT, PKR, Tat

## Abstract

Human immunodeficiency virus type 1 (HIV-1) has evolved various measures to counter the host cell's innate antiviral response during the course of infection. Interferon (IFN)-stimulated gene products are produced following HIV-1 infection to limit viral replication, but viral proteins and RNAs counteract their effect. One such mechanism is specifically directed against the IFN-induced Protein Kinase PKR, which is centrally important to the cellular antiviral response. In the presence of viral RNAs, PKR is activated and phosphorylates the translation initiation factor eIF2α. This shuts down the synthesis of both host and viral proteins, allowing the cell to mount an effective antiviral response. PACT (protein activator of PKR) is a cellular protein activator of PKR, primarily functioning to activate PKR in response to cellular stress. Recent studies have indicated that during HIV-1 infection, PACT's normal cellular function is compromised and that PACT is unable to activate PKR. Using various reporter systems and *in vitro* kinase assays, we establish in this report that interactions between PACT, ADAR1 and HIV-1-encoded Tat protein diminish the activation of PKR in response to HIV-1 infection. Our results highlight an important pathway by which HIV-1 transcripts subvert the host cell's antiviral activities to enhance their translation.

## Introduction

Cells infected with a virus employ a variety of mechanisms to counteract the negative impact of viral replication and promote cell survival [1]. The innate immune response to a viral infection is mediated by external and internal sensor molecules, which recognize the viral components as ‘non-self’ and trigger mechanisms leading to the production of interferons (IFNs) [2]. IFNs are secreted antiviral cytokines that bind in a paracrine and autocrine manner to cellular receptors and trigger signaling cascades culminating in the expression of IFN-stimulated genes (ISGs) [3]. Most ISGs have antiviral functions, although some ISGs with both antiviral and proviral functions have been previously described [4–6]. Viral and cellular factors regulate ISGs to promote or limit viral replication, respectively, and this regulatory interplay between the virus and the host cell is crucial in determining the outcome of a viral infection. Retroviruses such as the **h**uman **i**mmunodeficiency **v**irus type 1 (HIV-1) produce viral factors that interact with various cellular proteins, including ISGs. As a result, the virus subverts their antiviral properties or co-opts them from their regular cellular activities to facilitate efficient viral replication within the infected host cell [7,8].

One of the ISG products is PKR (**p**rotein **k**inase, **R**NA-activated), a protein kinase that plays a central role in regulating the outcome of a viral infection [9–11]. In virally infected cells, PKR is activated by binding to dsRNA (double-stranded RNA), a product of several viral infections, including HIV-1 [12,13]. The interaction between PKR and dsRNA induces a conformational change that is essential for PKR's catalytic activation [14]. PKR then phosphorylates the translation initiation factor eIF2α on serine 51, resulting in a decline of general protein synthesis, and consequent cessation of viral protein synthesis [15,16]. To counteract PKR's antiviral actions, viruses have developed measures that include dsRNA sequestration, decoy substrates, and direct interaction of virally encoded inhibitory proteins with PKR [17,18]. One of the host proteins that inhibits PKR activation during HIV-1 replication is the **T**AR **R**NA-**b**inding **p**rotein (TRBP), which was first identified due to its strong binding affinity for the ***t****rans*-**a**ctivation **r**esponse (TAR) element RNA found in the 5′-end of all HIV-1 mRNA transcripts [19,20]. In eukaryotic mRNAs, the 5′-untranslated region (UTR) is critical for ribosome recruitment to the mRNA, start codon choice and control of translation efficiency. This dual inhibitory effect of TAR on translation has promoted the development of viral countermeasures in order to achieve efficient viral replication. During HIV-1 infection, TRBP inhibits PKR activation by sequestration of the activating TAR RNA and by direct interaction with PKR's two dsRNA-binding motifs [21–23]. Although TRBP is an effective inhibitor of PKR, HIV-1 has evolved additional mechanisms to more effectively block PKR activity and successfully replicate in infected cells [10,24].

In the absence of viral infections, basal levels of PKR are present in all cells [9]. In uninfected cells, PKR regulates responses to oxidative stress, endoplasmic reticulum stress and serum starvation [25,26]. Under these conditions, a cellular **p**rotein **act**ivator of PKR (PACT) regulates PKR activation [27,28]. PACT is constitutively phosphorylated on serine 246 and is phosphorylated on serine 287 in response to stress, resulting in increased homodimerization and PACT–PKR heterodimerization [29–31]. PACT activates PKR and general protein synthesis is halted, allowing the cell to mount an effective response to the stressor or undergo apoptosis if the stressful conditions cannot be overcome. This stress response pathway is negatively regulated by TRBP, as TRBP interacts efficiently with PACT in the absence of stress. PACT's phosphorylation at serine 287 in response to cellular stress decreases its interaction with TRBP, and consequently, PACT–PACT and PACT–PKR interactions increase to activate PKR [31,32]. Thus, TRBP negatively regulates PKR activation, during viral infections and in response to cellular stress [33].

Recent studies established that PACT's function as a PKR activator is suppressed during HIV-1 infection, and PACT is unable to activate PKR in HIV-1-infected cells [10,24,34]. During the course of HIV-1 infection, there is a transient increase in PKR activation followed by a gradual decrease, which indicates the presence of a viral mechanism to subvert sustained PKR activation. A significant increase in the interactions between PACT, PKR and ADAR1 (**a**denosine **d**eaminase **a**cting on **R**NA **1**) is also observed and strongly correlates with decreased PKR activation and increased viral protein production [34]. The ADAR1-p150 isoform is an ISG-encoded, RNA-editing enzyme that catalyzes the deamination of adenosine to inosine in viral and cellular dsRNA substrates [35,36]. This often results in the destabilization of RNA secondary structures or incorporation of amino acids detrimental to viral protein structure and function [4,6,37]. In the present study, we further characterized the molecular mechanisms involved in mediating PACT's proviral effects during HIV-1 replication. Our findings indicate that PACT increases HIV-1 gene expression at the translational level via inhibition of PKR activation by acting in concert with an HIV-1-encoded protein Tat and a cellular protein ADAR1 to bring about sustained PKR inhibition and efficient translation of TAR-containing mRNAs. The present study underscores the essential role of Tat protein in this inhibitory complex and indicates that Tat enhances the translation of HIV-1 mRNAs in addition to its canonical trans-activation function during transcription [38] and its role as an inhibitory PKR pseudosubstrate [39]. Our study also highlights the importance of ADAR1 in this multiprotein complex as a key component that mediates PKR inhibition during HIV-1 infection. As all HIV-1 mRNAs contain the TAR structure at their 5′-end, these results shed light on how these mRNAs are efficiently translated in virally infected cells.

## Materials and methods

### Cell lines and antibodies

HeLa-MAGI-CCR5 cells [40] were obtained through the NIH AIDS Reagent Program. HeLa-MAGI-CCR5 cells, PKR^−/−^ murine embryonic fibroblasts (MEFs) [41], HEK-293T (ATCC CRL-11268) and HeLa (ATCC CRM-CCL-2) cells were cultured in Dulbecco's modified Eagle's medium (DMEM) containing 10% fetal bovine serum and penicillin/streptomycin. The following antibodies were used: anti-Flag monoclonal M2 (Sigma), anti-PKR (human) monoclonal (71/10, R&D Systems), anti-V5 (Invitrogen) and anti-Myc (Santa Cruz).

### Plasmids

The CMV-TAR-luciferase (CMV-TAR-LUC)/pGL3 basic plasmid was constructed as follows: the TAR sequence was inserted as an oligonucleotide in the *HindIII–BamHI* sites of pcDNA3-EGFP (Addgene). An 818 bp region containing the CMV promoter followed by TAR was excised from the TAR pcDNA3-EGFP plasmid described previously and inserted into the pGL3 basic vector (Promega) at the *SmaI–XhoI* site upstream of the luciferase-coding sequence. The corresponding CMV-luciferase pGL3 basic plasmid devoid of TAR was constructed as follows: a 753 bp region was excised from the pcDNA3-EGFP plasmid and inserted into the *SmaI–XhoI* site upstream of the luciferase-coding sequence. The mutant CMV-TAR-LUC/pGL3 basic was constructed by inserting the mutated TAR oligonucleotide into the *HindIII–BamHI* sites of pCDNA3-EGFP. The 818 bp region containing the CMV promoter and mutant TAR sequence was subsequently excised from the pcDNA3-EGFP expression construct and inserted into the pGL3 basic vector at the *SmaI–XhoI* site upstream of the luciferase-coding sequence. PACT and TRBP expression constructs were as described previously [29,32]. The Tat/pcDNA3 expression construct was a gift from Dr Ashok Chauhan (University of South Carolina) [42], while the pCMV-Rev and pcDNA3.1-ADAR1-p150-V5 expression constructs were previously described [43,44]. pCMV2-Flag-PACT was also previously described [32]. These constructs were a gift from Dr Anne Gatignol (McGill University).

### β-Galactosidase assay

HeLa-MAGI-CCR5 cells were transfected with indicated amounts of the Tat/pcDNA3, Flag PACT/pcMV2, Flag TRBP/pcDNA 3.1^−^ or only pcDNA 3.1^−^ expression constructs. β-Galactosidase activity was assayed 24 h after transfection using the Galacto-Star Assay System (ThermoFisher Scientific).

### Semi-quantitative reverse transcriptase PCR

Total RNA was isolated from HeLa-MAGI-CCR5 cells transfected with indicated amounts of the Tat/pcDNA 3, Flag PACT/pcMV2, Flag TRBP/pcDNA 3.1^−^ or pcDNA 3.1 expression constructs. After two washes with ice-cold PBS, 250 µl of RNAZol B was added and total RNA was isolated as per the manufacturer's instructions. cDNA was synthesized at 42°C for 1 h using random hexamer primers, 1 µg of total RNA, M-MuLV reverse transcriptase, 500 µM dNTPs and RNase inhibitor RNAsin (Promega). For each PCR, 2 µl of cDNA and 50 pmoles of forward and reverse primers designed to amplify a 166 bp region of the β-galactosidase transcript or a 500 bp region of the β-actin transcript were used with the Promega GoTaq Polymerase Kit. The following conditions were used for PCR: 95°C for 5 min (initial denaturation), denaturation at 95°C for 30 s, annealing at 45°C for 30 s and extension at 72°C for 30 s for 20, 25 or 30 cycles.

### Real-time PCR

RNA was isolated from PKR^−/−^ MEFs transfected with either the CMV-TAR-LUC pGL3 Basic or CMV-Luciferase pGL3 Basic plasmids and the indicated combinations of Flag wild-type PKR/pcDNA 3.1^−^, Tat/pcDNA 3 and Flag PACT/pCMV2 expression constructs using RNAzol B as per the manufacturer's instructions. DNAse treatment was performed to remove plasmid DNA from isolated RNA using the DNA-free™ DNAse Removal Kit (Ambion). cDNA was synthesized as described above using random hexamer primers. Real-time PCRs were performed with serial dilutions of cDNA to ensure efficiency. Reactions were performed in triplicate in a total reaction volume of 20 µl and included 4 µl of cDNA, 250 nM of firefly luciferase or β-actin primers and the SensiFAST™ SYBR® No-ROX Kit (Bioline). All reactions were run on a BioRad CFX96 Real-Time System C1000 thermal cycler machine with the following conditions: 95°C for 30 s, 95°C for 5 s, 53°C for 30 s (steps were repeated for 35 cycles), 60°C for 5 s and then 95°C for 5 s. We used the BioRad CFX Manager software to generate standard curves to compare luciferase expression in each sample. Two separate RNA isolations from transfected PKR^−/−^ MEFs were used for analysis.

### Transfections for luciferase reporter assays and real-time PCR analysis

All transfections were carried out in triplicate for each sample using indicated cell types cultured in six-well plates using Effectene (Qiagen) transfection reagent and 500 ng of total DNA per well. One nanogram of pRL-null (Promega) plasmid was co-transfected for normalization of the transfection efficiencies. Cell extracts were prepared at indicated time points, and firefly and Renilla luciferase activities were measured with the Dual Luciferase Reporter Assay system (Promega).

### PKR kinase activity assay

PKR activity assays were performed using an anti-PKR monoclonal antibody (71/10, R&D systems). HeLa cells were maintained in DMEM containing 10% fetal bovine serum. The cells were harvested when they were at 70% confluence. Cells were washed in ice-cold PBS and collected by centrifugation at 600 ***g*** for 5 min. Cell extracts were prepared in lysis buffer [20 mM Tris–HCl (pH 7.5), 5 mM MgCl_2_, 50 mM KCl, 400 mM NaCl, 2 mM dithiothreitol (DTT), 1% Triton-X 100, 100 U/ml aprotinin, 0.2 mM phenylmethylsulfonyl fluoride (PMSF) and 20% glycerol]. A 100 µg aliquot of total protein was immunoprecipitated using the anti-PKR monoclonal antibody (71/10) in high-salt buffer [20 mM Tris–HCl (pH 7.5), 50 mM KCl, 400 mM NaCl, 1 mM EDTA, 1 mM DTT, 1% Triton-X 100, 100 U/ml aprotinin, 0.2 mM PMSF and 20% glycerol] at 4°C for 30 min on a rotating wheel. A 20 µl aliquot of Protein-A agarose beads was then added and incubated for 1 h. The Protein-A agarose beads were washed four times in 500 µl of high-salt buffer and twice in activity buffer [20 mM Tris–HCl (pH 7.5), 50 mM KCl, 2 mM MgCl_2_, 2 mM MnCl_2_, 0.1 mM PMSF and 5% glycerol]. The PKR assay was performed with PKR still attached to the beads in activity buffer containing 0.1 mM ATP and 1 µCi of [γ^32^P] ATP at 30°C for 10 min. PKR was activated using synthesized TAR RNA (IDT DNA Technologies), and the effect of PACT, Tat, ADAR1 and TRBP on TAR-activated PKR was assayed by the subsequent addition of increasing amounts of pure recombinant PACT or pure recombinant TRBP (4, 40, 400 pg and 4 ng) in the presence of recombinant Tat and increasing amounts of recombinant ADAR1 (1.5, 15 and 150 ng). Labeled proteins were analyzed by SDS–PAGE on a 12% gel followed by autoradiography.

### Co-immunoprecipitation assay

*In vitro* translated, ^35^S-labeled ADAR1 and flag epitope-tagged PACT and TRBP proteins were synthesized using the TNT T7-coupled reticulocyte system from Promega. A 5 µl aliquot of ^35^S-labeled proteins was mixed in indicated combinations and incubated with 20 µl of anti-Flag mAb-agarose (Sigma) in 200 µl of immunoprecipitation (IP) buffer [20 mM Tris–HCl (pH 7.5), 150 mM (or 300 mM) NaCl, 1 mM DTT, 100 U/ml aprotinin, 0.2 mM PMSF, 20% glycerol and 1% Triton X-100] at room temperature for 30 min on a rotating wheel. The beads were washed in 500 µl of IP buffer four times and the washed beads were then boiled in Laemmli buffer [150 mM Tris–HCl (pH 6.8), 5% SDS, 5% β-mercaptoethanol and 20% glycerol] for 2 min and eluted proteins were analyzed by SDS–PAGE on a 12% gel followed by phosphorimager analysis for quantification.

### Yeast two-hybrid interaction assay

To compare the strength of TRBP–ADAR1 with PACT–ADAR1 interactions, ADAR1 was expressed as a GAL4 DNA-activation domain fusion protein from the pGADT7 vector, and TRBP and PACT were expressed as GAL4 DNA-binding domain fusion proteins from the pGBKT7 vector. ADAR1 pGADT7/TRBP pGBKT7 and ADAR1 pGADT7/PACT pGBKT7 were co-transformed into AH109 yeast cells (Clontech), and the transformed yeast cells were plated on double dropout SD (synthetic defined) minimal medium lacking tryptophan and leucine. To check for the transformants' ability to grow on triple dropout media, transformed yeast cells were grown to an OD_600_ of 2 in liquid growth medium. A 500 µl aliquot of each culture was pelleted and resuspended in an appropriate amount of distilled water to yield an OD_600_ of 10. Serial dilutions were then made to yield OD_600_ values of 1, 0.1 and 0.01. A 10 µl aliquot of each dilution was then spotted onto triple dropout SD minimal media lacking histidine, tryptophan and leucine. Plates were incubated at 30°C for 3 days.

### Quantifications and statistics

Radioactive bands were scanned for the TRBP–ADAR1 and PACT–ADAR1 co-immunoprecipitation assays (Typhoon FLA7000) were quantified using the GE Life Sciences ImageQuant TL software. To determine the statistical significance of the results of the co-immunoprecipitation assay and the β-galactosidase and luciferase assays, a two-tailed Student's *t*-test was performed, assuming equal variance. Each figure legend indicates *P*-values as denoted by brackets and special characters. Note that our α-level was *P* = 0.05.

## Results

### PACT enhances HIV-1 gene expression from a Tat-induced integrated long terminal repeat

**Figure 1.**
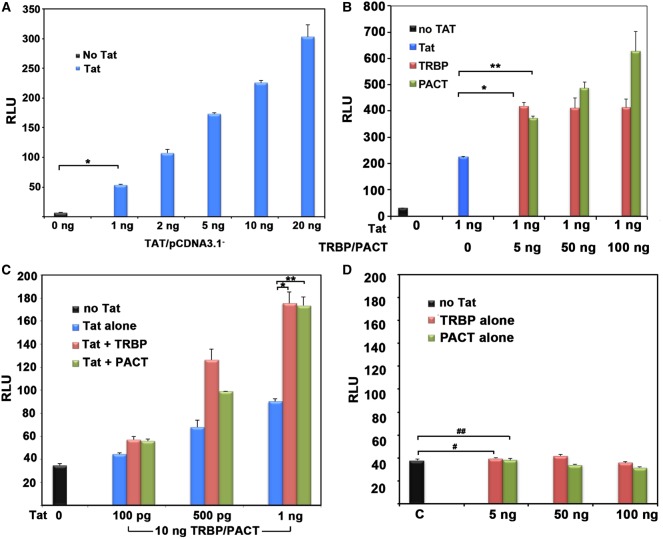
PACT activates Tat-enhanced HIV-1 gene expression from an integrated LTR. (**A**) Requirement and dose–response curve for Tat for HIV-1 LTR-driven expression. HeLa-MAGI-CCR5 cells were transfected with 0 (black bar) or increasing amounts of Tat/pcDNA 3 (blue bars) as indicated. β-Galactosidase activity was assayed 24 h after transfection. Error bars indicate standard deviation calculated from three independent experiments. The *P*-value (0.0000038) calculated using statistical analyses indicated significant difference between the RLU values indicated by the bracket marked as ‘*’. (**B** and **C**) PACT and TRBP both enhance HIV-1 LTR-driven expression. (**B**) HeLa-MAGI-CCR5 cells were transfected with 0 (black bar) or 1 ng Tat/pcDNA 3 (blue bar), with increasing amounts of Flag TRBP/pcDNA 3.1^−^ (red bars) or Flag PACT/pCMV2 (green bars) as indicated. The *P*-values (0.0045 and 0.0044) calculated using statistical analyses indicated significant difference between the RLU values indicated by the brackets marked as ‘*’ and ‘**’, respectively. (**C**) HeLa-MAGI-CCR5 cells were transfected with 0 ng, (black bar), increasing amounts of Tat/pcDNA 3 (blue bars) and with 10 ng of Flag TRBP/pcDNA 3.1^−^ (red bars) or with Flag PACT/pCMV2 (green bars). β-Galactosidase activity was assayed 24 h post-transfection. Error bars indicate the standard deviation calculated from three independent experiments. The *P*-values (0.0011 and 0.0004) calculated using statistical analyses indicated significant difference between the RLU values indicated by the brackets marked as ‘*’ and ‘**’, respectively. (**D**) PACT and TRBP have no effect on HIV-1 LTR-driven expression in the absence of Tat. HeLa-MAGI-CCR5 cells were transfected with 0 (black bar), or increasing amounts of Flag TRBP/pcDNA 3.1^−^ (red bars) or Flag PACT/pCMV2 (green bars). β-Galactosidase activity was assayed 24 h post-transfection. All transfections were compensated to the same amount of DNA with pcDNA 3.1. Error bars indicate standard deviation calculated from three independent experiments. The *P*-values (0.3863 and 0.9124) calculated using statistical analyses indicated no significant difference between the RLU values indicated by the brackets marked as ‘#’ and ‘##’, respectively.

Our previous work indicated that PACT enhances expression from a HIV-1 promoter in transfected HeLa cells and viral replication in HIV-1-infected cells [32,34]. Thus, in this context, PACT exhibited a proviral function similar to the PKR inhibitor TRBP. To determine if PACT can enhance HIV-1 long terminal repeat (LTR)-driven gene expression in the context of latently infected cells, we first compared the effects of TRBP and PACT when HIV-1 LTR is integrated into the host chromosome. HeLa-MAGI-CCR5 cells contain a stably integrated β-galactosidase-coding region expressed under the control of the HIV-1 LTR, whose transcription is dependent on HIV-1 Tat protein [45–49]. We first verified that increasing amounts of Tat expression vector (blue bars) resulted in a dose-dependent increase in β-galactosidase activity (Figure 1A) compared with the absence of Tat (black bar). Having confirmed that the cells are responsive to Tat, we next evaluated the effect of PACT in comparison to TRBP. The addition of PACT (green bars) or TRBP (red bars) expression constructs further stimulated Tat-trans-activated HIV-1 LTR-driven β-galactosidase activity (Figure 1B). Furthermore, the addition of increasing amounts of Tat expression plasmid (blue bars) in the presence of a constant amount of PACT (green bars) or TRBP (red bars) led to increased β-galactosidase activity in a dose-dependent manner (Figure 1C). In contrast, in the absence of Tat, neither PACT (green bars) nor TRBP (red bars) had any effect on HIV-1 LTR-driven expression (Figure 1D). These results indicate that similar to TRBP, PACT activates expression from HIV-1 LTR when integrated in the host chromosome and that this effect is dependent on the presence of the viral Tat protein.

### PACT does not affect the steady-state transcript levels of HIV-1 LTR-driven genes

**Figure 2.**
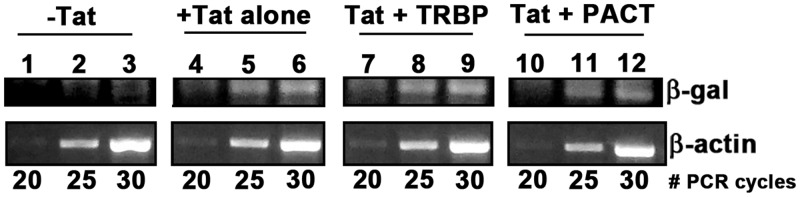
PACT does not increase the steady-state mRNA levels of HIV-1 LTR-driven β-galactosidase. RNA was isolated from HeLa-MAGI-CCR5 cells transfected with pcDNA 3.1^−^ only (−Tat, lanes 1–3), Tat/pcDNA 3 (+Tat alone, lanes 4–6), Tat pcDNA 3 and Flag TRBP/pcDNA 3.1^−^ (Tat + TRBP, lanes 7–9) or Tat/pcDNA 3 and Flag PACT/pCMV2 (Tat + PACT, lanes 10–12). The RNA preparation was treated extensively with DNase to digest any DNA contamination. β-Galactosidase mRNA expression levels were analyzed by semi-quantitative RT-PCR at the indicated number of reaction cycles. β-Actin mRNA levels were also analyzed as a normalization control for each cycle number. No PCR products were obtained in the absence of reverse transcriptase.

To characterize PACT's activating effect on HIV-1 LTR-driven gene expression as either transcriptional or post-transcriptional, we performed semi-quantitative RT-PCR analysis to assess changes in β-galactosidase mRNA levels in HeLa-MAGI-CCR5 cells transfected with Tat and PACT or TRBP expression plasmids relative to β-actin mRNA levels (Figure 2). As expected, we observed that the expression of Tat increased β-galactosidase mRNA levels (lanes 4–6) when compared with empty vector-transfected HeLa-MAGI-CCR5 cells (lanes 1–3). As seen in lanes 7–9, there was no increase in β-galactosidase mRNA levels between HeLa-MAGI-CCR5 cells transfected with Tat alone (lanes 4–6) and HeLa-MAGI-CCR5 cells transfected with Tat and TRBP (lanes 7–9) or Tat and PACT (lanes 10–12). These results show that the enhancing effect of PACT on β-galactosidase activity in HeLa-MAGI-CCR5 cells was not a result of increased levels of β-galactosidase mRNA, but resulted from a post-transcriptional mechanism. As all mRNAs produced from HIV-1 LTR promoter-driven reporters contain a TAR structure in their 5′-UTRs, these results indicate that PACT acts at a post-transcriptional level on TAR-containing mRNAs.

### Tat and PACT inhibit PKR activation induced by TAR-containing mRNAs

The translation of HIV-1 mRNAs is diminished by the TAR RNA secondary structure in their 5′-UTRs and also by TAR-mediated PKR activation [7,50,51]. This effect is partially compensated for by the cellular proteins TRBP and ADAR1 [22,43,52–54] as well as by the viral protein Tat [39]. However, Tat also acts as a potent transcriptional trans-activator for HIV-1 LTR-driven genes, and in order to specifically study Tat's post-transcriptional effects, we used a system that is not affected at the transcriptional level by Tat. For this purpose, we designed an expression construct CMV-TAR-LUC, in which the TAR RNA was placed directly upstream of the firefly luciferase open reading frame expressed from a CMV promoter. A CMV-Luciferase expression construct (CMV-LUC) was designed as a control without TAR. By producing TAR-containing transcripts from the CMV promoter which is nonresponsive to Tat's transcriptional trans-activation, we could specifically assess Tat's post-transcriptional effects mediated by PKR activation.

**Figure 3.**
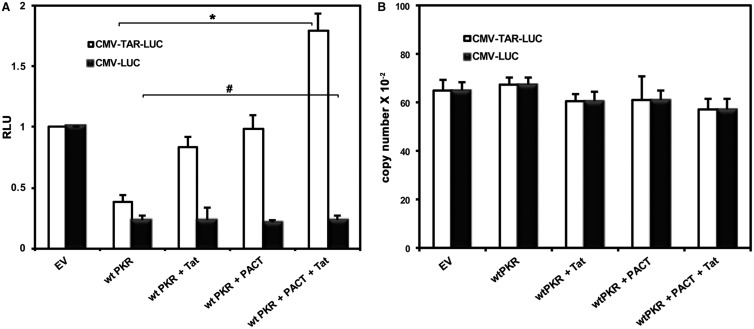
PACT inhibits PKR activation induced by TAR-containing mRNAs. (**A**) Tat enhances PACT-mediated PKR inhibition on TAR-containing mRNAs. PKR^−/−^ MEFs were co-transfected with CMV-TAR-luciferase or CMV luciferase (in pGL3 basic plasmid backbone), and indicated combinations of Tat/pcDNA3, Flag PKR/pcDNA 3.1^−^ and Flag PACT/pCMV2. Firefly luciferase activity was assayed 24 h post-transfection and the error bars represent the standard deviation from three experiments. The *P*-values (0.0018 and 0.4601) calculated using statistical analyses indicated significant and non-significant differences between the RLU values indicated by the brackets marked as ‘*’ and ‘#’, respectively. (**B**) qRT-PCR analysis. qRT-PCR analysis was performed to analyze luciferase mRNA expression levels in samples from PKR^−/−^ MEFs transfected as described in **A**. Data represent the average from six replicate experiments from two different RNA isolations. All results are normalized to β-actin.

To examine the activity of PACT and Tat on PKR-induced inhibition of translation, PKR^−/−^ MEFs were co-transfected with either CMV-TAR-LUC/pGL3 Basic or CMV-Luciferase/pGL3 Basic along with PACT and Tat expression plasmids, and luciferase activity was assessed. As seen in Figure 3A, co-transfection of PKR with CMV-TAR-LUC (white bars) or CMV-LUC (black bars) reduced luciferase activity as previously reported and in agreement with PKR's effect on translation of plasmid-encoded transcripts [22,32,34,43]. Furthermore, co-transfection of Tat with PKR rescued the PKR-mediated reduction in luciferase activity only when TAR was present, indicating that Tat can relieve the translational block imposed by PKR on TAR-containing mRNAs. Surprisingly, PACT also counteracted TAR-induced PKR translational inhibition, whereas it maintained PKR-mediated translational inhibition of plasmid-derived luciferase mRNA in the absence of TAR, suggesting that the presence of TAR is required for both PACT and Tat's inhibitory effect on PKR. In addition, when expressed together, PACT and Tat showed a further significant increase in luciferase expression with CMV-TAR-LUC, but not with CMV-LUC. These results indicate that PACT inhibits PKR activation on TAR-containing mRNAs, in contrast with its well-characterized PKR-activating function [27,28,55–57]. Furthermore, this PKR inhibitory activity of PACT can only occur in the presence of TAR-containing mRNA transcripts (compare black bars with white bars) and is significantly enhanced in the presence of Tat. These results suggest that during active production of HIV-1 viral proteins, PACT acts in concert with Tat and TAR RNA to counteract PKR-mediated inhibition of viral mRNA translation.

To ensure that the Tat-dependent, PACT-mediated inhibition of PKR was not a result of changes in TAR-firefly luciferase mRNA or firefly luciferase mRNA transcript levels, we performed qRT-PCR analysis to quantify firefly luciferase mRNA levels in total RNA isolated from the PKR^−/−^ MEFs transfected with the constructs indicated in Figure 3A. There were no significant differences in firefly luciferase mRNA levels in the various samples (Figure 3B), demonstrating that PACT's Tat-dependent effect on luciferase expression is at the translational level, most probably by counteracting PKR activation.

### Tat–TAR interaction is essential for PACT's PKR inhibitory activity

**Figure 4.**
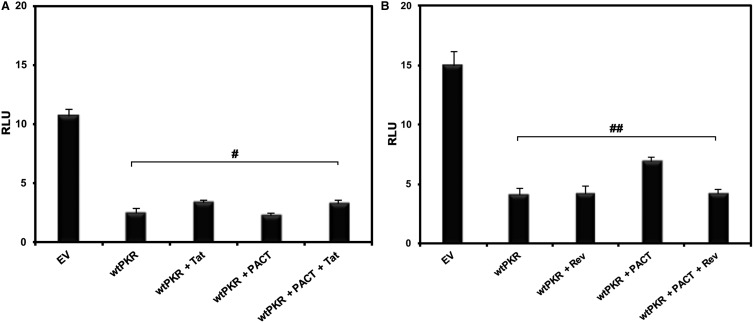
Tat serves a specific function in mediating inhibition of PKR. (**A**) Tat's binding to the TAR is essential for PACT-mediated PKR inhibition. PKR^−/−^ MEFs were co-transfected with CMV mutant TAR-luciferase (in pGL3 basic plasmid backbone: EV) in which the mutant TAR element does not bind to Tat and the indicated combinations of Tat/pcDNA 3, Flag PKR/pcDNA 3.1^−^ and Flag PACT/pCMV2. Firefly luciferase activity was assayed 24 h post-transfection and the error bars represent the standard deviation from three experiments. The *P*-value (0.5852) calculated using statistical analyses indicated a non-significant difference between the RLU values indicated by the bracket marked as ‘#’. (**B**) Rev, another HIV-1-encoded RNA-binding protein, cannot substitute for Tat's function. PKR^−/−^ MEFs were co-transfected with the CMV-TAR-luciferase expression construct and the indicated combinations of Rev/pcDNA 3, Flag PKR/pcDNA 3.1^−^ and Flag PACT/pCMV2. Luciferase activity was assayed 24 h after transfection and the error bars represent the standard deviation from three experiments. The *P*-value (0.5852) calculated using statistical analyses indicates no significant difference between the RLU values indicated by the bracket marked as ‘##’.

As we observed that Tat and PACT work synergistically to increase translation of TAR-containing mRNAs, we wished to determine if Tat's ability to bind to the TAR RNA was essential for this function. To test this, we generated a CMV-TARm-LUC construct in which the TARm RNA will not bind Tat but would still activate PKR [58]. PKR co-transfection with this construct dramatically reduced the firefly luciferase activity (Figure 4A). However, co-transfection of Tat or PACT had no effect on PKR-mediated inhibition of the luciferase activity. These results show that Tat's ability to interact with TAR-containing mRNAs is essential for the concerted PKR inhibitory effect of Tat and PACT on TAR-containing mRNAs.

To test that the PKR inhibitory effect was specific to Tat–PACT combination, we performed the same transfection experiments with CMV-TAR-LUC and Rev, which is an HIV-1 viral protein that binds to a different structured RNA element in viral mRNAs known as the Rev response element [59–61]. Co-transfection of PKR reduced the luciferase activity as shown in Figure 3A, but that of PACT or/and Rev had no effect on luciferase activity (Figure 4B). These results confirm that PACT's PKR inhibitory activity on TAR-containing mRNAs specifically requires Tat and its TAR RNA-binding activity is essential for this function.

### Additional cellular factors are essential to inhibit TAR RNA-mediated PKR activation

**Figure 5.**
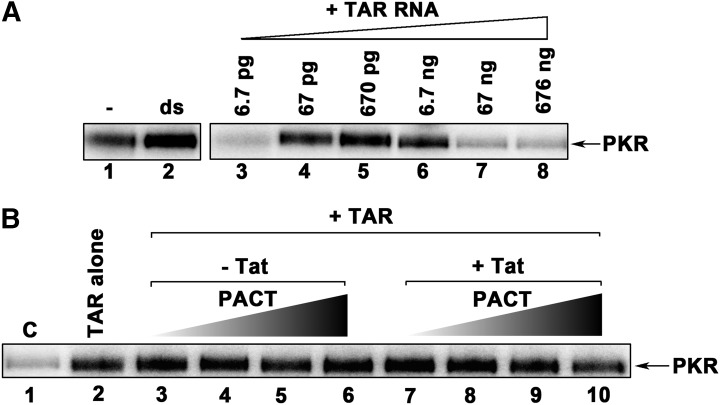
PACT and Tat are insufficient to inhibit TAR RNA-dependent PKR activation. (**A**) TAR RNA activates PKR: PKR immunoprecipitated from HeLa cell extracts was activated by the addition of increasing amounts of TAR RNA as indicated (lanes 3–8) or poly I:poly C (lane 2, ds). Lane 1 indicates activity in the absence of any activator. The phosphorylated proteins were analyzed by SDS–PAGE and phosphorimager analysis. (**B**) Efficient inhibition of PKR requires components in addition to PACT and Tat. PKR immunoprecipitated from HeLa cell extracts was activated with 67 pg of TAR RNA (lanes 2–10). Increasing amounts of pure recombinant PACT (lanes 3–6 and lanes 7–10) were added in the absence of Tat (lanes 3–6) or in combination with 4 ng of pure recombinant Tat (lanes 7–10). PACT amounts are as follows: 4 pg (lanes 3 and 7), 40 pg (lanes 4 and 8), 400 pg (lanes 5 and 9) and 4 ng (lanes 6 and 10). Lane 1 (C) shows the PKR activity without any added activator. The phosphorylated proteins were analyzed by SDS–PAGE and phosphorimager analysis.

Based on the above results, the combination of the TAR RNA, PACT and Tat seems to induce strong inhibition of PKR activation in cell culture. To determine if those components are sufficient to provide complete PKR inactivation, we performed *in vitro* kinase activity assays using PKR immunoprecipated from HeLa cells to recapitulate the mechanism *in vitro*. We first confirmed that PKR is activated robustly by TAR RNA similar to the synthetic dsRNA polyI:C, with a bell-shaped activation curve with no activation at low and high concentrations of TAR RNA (Figure 5A), as previously observed [62–64]. We then assessed PACT's ability to inhibit or activate PKR activation caused by TAR RNA in the absence (Figure 5B, lanes 3–6) or presence (Figure 5B, lanes 7–10) of Tat. PACT remained a PKR activator in the presence of TAR RNA (Figure 5B, lanes 3–6). In the presence of Tat, very modest inhibition of PKR activity with the highest amount of PACT was observed (Figure 5B, lane 10), indicating that PACT and Tat cannot recapitulate PKR inhibition *in vitro* and that additional components present in mammalian cells are required for the observed inhibition of PKR activation on TAR-containing mRNAs.

### The RNA-editing protein ADAR1 is essential for complete inhibition of TAR-activated PKR by Tat and PACT

**Figure 6.**
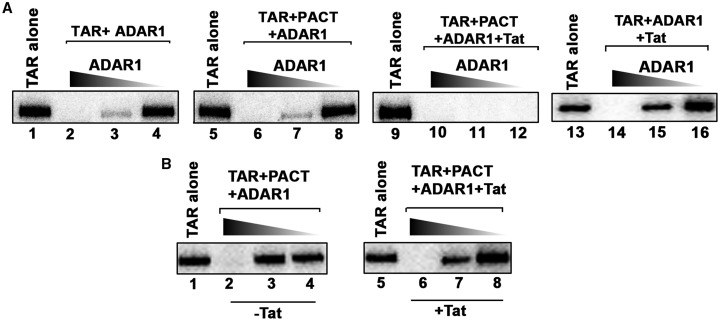
ADAR1 is essential for an efficient inhibition of PKR. (**A**) ADAR1, PACT and Tat inhibit PKR efficiently. PKR immunoprecipitated from HeLa cell extracts was activated with 67 pg of TAR RNA and varying amounts (150, 15 and 1.5 ng) of ADAR1 (lanes 2–4, 6–8, 10–12 and 14–16) were added as indicated to assess the inhibition of PKR. Lanes 2–4: PKR activity in the presence of varying amounts of ADAR1 alone; lanes 6–8: PKR activity in the presence of 4 ng of PACT and varying amounts of ADAR1; lanes 10–12: PKR activity in the presence of 4 ng of PACT, 4 ng of Tat and varying amounts of ADAR1; lanes 14–16: PKR activity in the presence of 4 ng of Tat and varying amounts of ADAR1. The phosphorylated proteins were analyzed by SDS–PAGE and phosphorimager analysis. (**B**) ADAR1 and PACT inhibit PKR more efficiently in the presence of Tat. PKR immunoprecipitated from HeLa cell extracts was activated with 67 pg of TAR RNA and varying amounts (1.5 ng, 150 pg and 15 pg) of ADAR1 (lanes 2–4 and 6–8) were added as indicated to assess the inhibition of PKR. Lanes 2–4: PKR activity in the presence of varying amounts of ADAR1 and 4 ng of PACT; lanes 6–8: PKR activity in the presence of varying amounts of ADAR1, 4 ng of PACT and 4 ng of Tat. The phosphorylated proteins were analyzed by SDS–PAGE and phosphorimager analysis.

We previously reported that another dsRNA-binding protein, ADAR1, directly interacts with PKR and PACT during HIV-1 infection to form a PKR inhibitory complex [24,34,43]. Thus, we investigated if ADAR1 can inhibit TAR-activated PKR when present together with Tat and PACT. Using an *in vitro* kinase assay, we observed that ADAR1 can inhibit PKR activation efficiently in a dose-dependent manner (Figure 6, lanes 2–4). Under these conditions, 150 ng of ADAR1 was required for complete inhibition of PKR activity (lane 2), whereas 15 and 1.5 ng of ADAR1 showed partial (lane 3) and no inhibition (lane 4), respectively. The addition of PACT did not improve or compromise the PKR inhibitory function of ADAR1 (lanes 6–8). We then tested the effect of HIV-1 Tat protein on PKR activity as our results in Figure 3 suggested that Tat is required for PKR inhibition. When Tat was present, we observed a complete inhibition of PKR activity at all concentrations of ADAR1 (lanes 9–12). Thus, the Tat protein seems to significantly enhance the PKR inhibitory actions of ADAR1; 100-fold less ADAR1 (lane 12, 1.5 ng ADAR1) was sufficient to inhibit PKR activity in the presence of Tat, when compared with the conditions where Tat was absent (lane 6, 150 ng ADAR1). Also, Tat when present with ADAR1 does not enhance ADAR1's PKR inhibitory actions when compared with the inhibition observed with ADAR1 alone (lanes 14–16) when PACT is absent. These results show that Tat, PACT and ADAR1 act in concert to inhibit PKR and suggest that an inhibitory complex formed with Tat, PACT and ADAR1 is essential for efficient PKR inhibition on TAR-containing HIV-1 mRNAs. Tat by itself (data not shown) or with PACT (Figure 5) does not inhibit PKR activity. To further confirm that Tat enhances the PKR inhibitory activity of ADAR1 and PACT, we compared the effect of lower concentrations of ADAR1 in the presence and absence of Tat. As seen in Figure 6B, in the absence of Tat, 1.5 ng of ADAR1 showed complete inhibition of PKR activity (lane 2), and 150 and 15 pg ADAR1 showed no PKR inhibition (lanes 3 and 4, respectively). In the presence of Tat, 1.5 ng of ADAR1 showed complete inhibition (lane 6) and 150 pg of ADAR1 showed partial inhibition of PKR activity (lane 7). These results demonstrate that Tat enhances the PKR inhibitory actions of ADAR1 in the presence of PACT, and that Tat may function to recruit PACT and ADAR1 to the complex after binding to TAR in HIV-1-encoded transcripts.

### A comparison of PKR inhibitory activity of ADAR1 in the presence of PACT or TRBP

**Figure 7.**
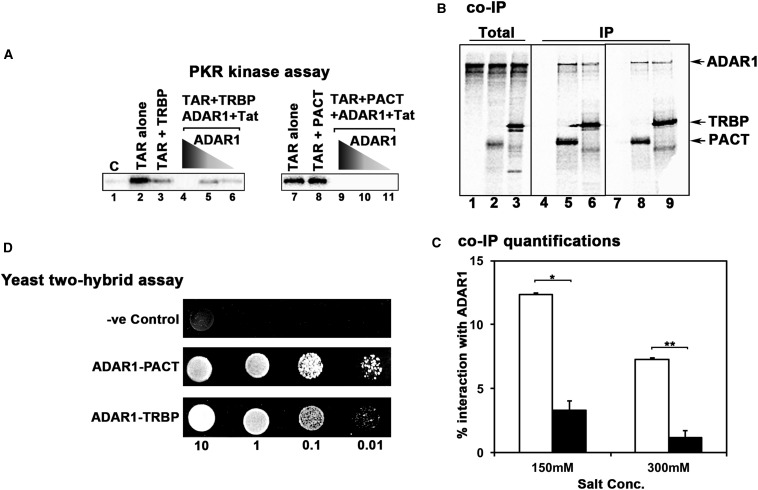
Comparison of TRBP and PACT's PKR inhibitory activity on TAR RNA. (**A**) PACT is more efficient compared with TRBP in forming a PKR inhibitory complex. PKR immunoprecipitated from HeLa cell extracts was activated with 67 pg of TAR RNA. Either 4 ng of PACT (lane 2) or TRBP (lane 8) or 4 ng of TRBP (lanes 4–6) or PACT (lanes 9–11), 4 ng of Tat and varying amounts (150, 15 and 1.5 ng) of ADAR1 (lanes 4–6 and 9–11) were added as indicated to assess the inhibition of PKR. Lanes 4–6 represent PKR activity in the presence of 4 ng of TRBP. Lanes 9–11 represent PKR activity in the presence of 4 ng of PACT. Lane 1 (C) shows the PKR activity without any added activator. The phosphorylated proteins were analyzed by SDS–PAGE and phosphorimager analysis. (**B**) PACT interacts with ADAR1 with higher affinity when compared with TRBP. A 5 µl aliquot of *in vitro* translated, ^35^S-labeled Flag-tagged PACT and TRBP proteins was mixed with 5 µl of *in vitro* translated, ^35^S-labeled ADAR1. Flag PACT proteins were immunoprecipitated using anti-Flag mAb-agarose, and ADAR1 co-immunoprecipitation was analyzed by SDS–PAGE. Total: input (20% of the IP samples); IP: immunoprecipitates. The bands seen at lower positions than the TRBP band in lanes 3, 6 and 9 are truncated TRBP proteins produced by translation initiation at internal methionines in the reticulocyte lysate system. (**C**) Quantification of data in **B**. The radioactivity present in the bands was measured by phosphorimager analysis and the % co-IP was calculated as follows: (radioactivity present in the co-immunoprecipitated ADAR1 band/the radioactivity present in the ADAR1 band in the total lane) × 100. This value was normalized to the amount of radioactivity present in the PACT or TRBP bands in IP lanes to correct for differences in translation/immunoprecipitation. Error bars: standard deviation from three independent experiments. The *P*-values (0.000029 and 0.000051) calculated using statistical analyses indicate significant difference between % co-IP of ADAR1 with PACT (white bars) and TRBP (black bars) at 150 mM (*) and 300 mM (**) salt concentrations, respectively. (**D**) Yeast two-hybrid assay to compare TRBP–ADAR1 and PACT–ADAR1 interactions. TRBP or PACT in pGBKT7 and ADAR1 in PGADT7 or empty pGADT7 plasmids were co-transformed into AH109 yeast cells and selected on SD double dropout media lacking tryptophan and leucine. Aliquots (10 μl) of serial dilutions (OD_600_ = 10, 1.0, 0.1 and 0.01) were spotted for each transformant on a triple dropout SD medium plate that lacks tryptophan, leucine and histidine. Plates were incubated for 3 days at 30°C. Transformation of PACT or TRBP constructs in pGBKT7 and empty vector pGADT7 served as negative controls.

As TRBP is known to inhibit PKR under various conditions including HIV infection, we wished to compare the relative efficiency of PACT and TRBP to inhibit TAR RNA-activated PKR in the presence of Tat and ADAR1. As seen in Figure 7A, we observed that similar to PACT, ADAR1 can inhibit PKR activation efficiently in a dose-dependent manner in the presence of TRBP and Tat (lanes 4–6). Under these conditions, 150 ng of ADAR1 was required for a complete inhibition of PKR activity (lane 4). Unlike PACT (which activates PKR in the absence of ADAR1), TRBP shows significant inhibition of PKR even in the absence of ADAR1 (lane 3), and this inhibition is further enhanced by the addition of ADAR1 (lanes 4–6). Comparing the relative efficiency of TRBP and PACT to inhibit PKR, 100-fold less ADAR1 is required in the presence of PACT (lanes 9–11) when compared with conditions where TRBP was used (lanes 4–6) instead of PACT. Thus, PACT significantly enhances the PKR inhibitory actions of ADAR1 when compared with TRBP (lanes 4–6 and 9–11). These results show that Tat, PACT and ADAR1 act in concert to inhibit PKR more efficiently than Tat, TRBP and ADAR1. One possible mechanism for PACT's enhanced ability to increase ADAR1's effective inhibition of PKR could result from its higher affinity for ADAR1. Therefore, we compared the relative strengths of PACT–ADAR1 and TRBP–ADAR1 interactions using a co-immunoprecipitation assay. We used an *in vitro* rabbit reticulocyte translation system to generate ^35^S-methionine-labeled ADAR1, PACT and TRBP proteins. As seen in Figure 7B, both PACT and TRBP can co-immunoprecipitate ADAR1 at 150 mM (lanes 4–6) and 300 mM (lanes 7–9) salt concentrations. However, at both salt concentrations, PACT interacts significantly more efficiently with ADAR1 when compared with TRBP (Figure 7C). At 150 mM salt concentration, PACT pulled down 12.3% of ADAR1, whereas TRBP could only pull down 3.2% of ADAR1. At 300 mM salt concentration, PACT pulled down 7.3% of ADAR1 and TRBP pulled down only 1.1% of ADAR1. To compare the PACT–ADAR1 and TRBP–ADAR1 interactions further, we utilized a yeast two-hybrid assay (Figure 7D). We have used this system extensively to demonstrate that stress-induced phosphorylation of PACT results in changes in the affinity of its interaction with TRBP and PKR [29,31,65]. Thus, the yeast two-hybrid system is sensitive enough to detect changes in relative affinities between these proteins and measures direct interaction between two proteins. As seen in Figure 7D, in comparison with TRBP, PACT shows significantly stronger interaction with ADAR1. These results further suggest that ADAR1 functions as a more efficient inhibitor of TAR RNA-activated PKR in the presence of PACT than in the presence of TRBP, either because PACT recruits ADAR1 with higher efficiency to TAR-containing mRNAs or because ADAR1 forms a more stable PKR inhibitory complex with PACT. In HIV-infected cells, it is possible that both TRBP and PACT form complexes with Tat, ADAR1 and TAR RNA, but PACT functions more efficiently to bring about PKR inhibition.

## Discussion

In HIV-1-infected patients type I IFNs are produced by plasmacytoid dendritic cells and exert both antiviral and immunomodulatory activities [66]. However, this IFN response is insufficient to clear the virus from infected cells [67,68]. The inability of IFNs to clear the virus is not due to a lack of cellular response to IFN since the ISGs are induced in infected peripheral blood mononuclear cells (PBMCs) when in culture and show inhibition of HIV-1 replication [34]. Thus, the absence of a robust IFN antiviral response in patients is due to a block in the antiviral actions of ISGs. PKR is one of the ISGs whose regulation has been studied extensively in the context of many viral infections including HIV-1. PKR overexpression results in its activation that effectively restricts HIV-1 replication [21,43,69–71]. In addition, a knockdown of PKR using siRNAs or overexpression of a trans-dominant negative PKR mutant results in increased HIV-1 replication in cell culture [53]. In spite of this, the virus replicates efficiently in patient cells, suggesting that PKR activity is heavily limited during the course of a natural infection [24]. Our previous work showed that PKR activation takes place only transiently after HIV-1 infection of PBMCs or of lymphocytic cell lines with either X4 or R5 HIV-1 strains, suggesting that PKR activation is rapidly inhibited by the presence of HIV-1, which removes a barrier to replication [43]. During the course of HIV-1 infection, PKR is activated by the TAR RNA and inhibited by TRBP, ADAR1 and the viral Tat proteins. Of these inhibitors, the HIV-1 protein Tat inhibits PKR by acting as a substrate competitor [39,72,73], whereas TRBP and ADAR1 inhibit PKR activity by direct interaction. TRBP also sequesters the activator dsRNA and PACT molecules by a direct interaction with them [21,23]. ADAR1 was previously identified as an important contributor for effective PKR inhibition and has emerged as exhibiting both antiviral and proviral functions [4,10]. ADAR1 catalyzes the deamination of adenosine in RNAs with dsRNA regions, thereby causing a destabilization of RNA duplexes and genetic recoding [35]. Thus, ADAR1 functions as a suppressor of dsRNA-mediated antiviral responses, which include activation of PKR and IFN regulatory factor IRF3, the transcription factor for IFN genes [4]. The p150 isoform of ADAR1 is an ISG, present both in the cytoplasm and nucleus, while the p110 isoform is constitutively expressed and is predominantly present in the nucleus [74].

The results presented here demonstrate that TAR-mediated PKR activation is also suppressed by a complex of PACT, ADAR1 and the viral protein Tat. Thus, in addition to its well-established functions in the nucleus and in transcription of the HIV-1 proviral genome, Tat plays an important function in enhancing HIV-1 mRNA translation in the cytoplasm. Furthermore, in this complex, PACT is unable to activate PKR, and ADAR1 and Tat are essential for repressing PACT's canonical PKR-activating role. Neither Rev nor a mutated TAR can inhibit PKR activation, thus suggesting that TAR RNA serves as a scaffold to recruit and stabilize many RNA-binding proteins, and PACT's PKR-activating ability is inactivated by the recruitment of ADAR1 to this complex. It is possible that Tat binds to TAR first to recruit PACT, which in turn is able to efficiently bring ADAR1 to the complex. Our previous data established that PACT and ADAR1 interact directly [34], and our current results show that Tat has an essential function in this complex. Overall, our results suggest that during HIV-1 infection, cytoplasmic Tat may bind to the TAR RNA to simultaneously recruit PACT and ADAR1 to serve a PKR inhibitory role. This complex serves a crucial role in enhancing translation of viral proteins needed for efficient viral replication as PKR is known to bind to the stem region of TAR RNA [75].

**Figure 8.**
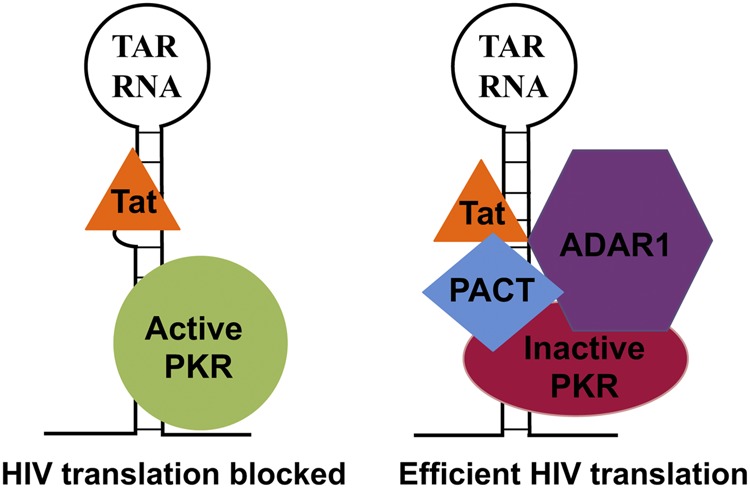
Schematic model. When TAR RNA present at the 5′-end of HIV-1-encoded transcripts binds PKR, activated PKR blocks translation of TAR-containing HIV-1 mRNAs. Efficient translation of HIV-1 viral proteins occurs by recruiting viral protein Tat and host factors PACT and ADAR1, to efficiently block PKR activation, thereby allowing synthesis of viral proteins. In this complex, the PKR-activating role of PACT is suppressed by the presence of ADAR1.

In addition to its classical transcriptional trans-activation role in the nucleus, Tat's cytoplasmic functions during HIV-1 replication have been reported before in other studies [76,77]. Tat protein counteracts the effect of TAR to stimulate translation of the viral mRNAs by enhancing the activity of RNA helicase DDX3 [78–81]. Tat also showed a stimulatory effect on global protein synthesis by competing with eIF2α for phosphorylation by PKR or by inhibiting PKR activity, independently of the presence of TAR (reviewed in ref. [24]). Our work introduces one more regulatory layer for Tat's central role in HIV-1 replication. As represented in Figure 8, our results establish that, for efficient translation of TAR-containing mRNAs, the interaction between TAR and Tat is essential to promote the formation of a PKR inhibitory complex that contains PACT and ADAR1. Using a mutated TAR region that does not bind Tat but can activate PKR efficiently, we demonstrate that PKR-mediated translational down-regulation was not overcome in the absence of TAR–Tat interaction (Figure 4). As a part of this multiprotein complex, PACT is unable to activate PKR and ADAR1 strongly represses PKR activity (Figure 6).

ADAR1 has been shown to inhibit PKR activity and reduce eIF2α phosphorylation efficiently to play a proviral role during the replication of several DNA and RNA viruses [4,6]. Overexpression of either the full-length ADAR1-p150 protein or the region with the RNA- and Z-DNA-binding domains alone inhibited PKR autophosphorylation and eIF2α phosphorylation [43,82]. A stable knockdown of ADAR1 expression causes enhanced PKR autophosphorylation and eIF2α phosphorylation following infection with measles virus or vesicular stomatitis virus [83,84]. In ADAR1-containing cells, PKR autophosphorylation is suppressed following viral infection, but in ADAR1-deficient cells it is enhanced because of the lack of editing-mediated destabilization of dsRNA, lack of sequestration of dsRNA by ADAR1 and also because of a lack of formation of inactive heterodimeric ADAR1:PKR complexes [4].

Furthermore, a depletion of ADAR1 by RNAi in human cells or by genetic knockout in mouse MEFs leads to enhanced apoptosis and cytotoxicity following infection with RNA viruses from the Paramyxoviridae and the Rhabdoviridae families as well as the *polyoma* DNA virus [83–86]. Using an overexpression screening strategy in which more than 380 human ISGs were tested for their antiviral activity against many medically important viruses, ADAR1 emerged as the most potent *proviral* ISG, which enhanced the replication of HIV-1, West Nile virus, Chikungunya virus, Venezuelan equine encephalitis virus and yellow fever virus [87]. In case of HIV-1 infection, our results demonstrate that ADAR1 is important to suppress PKR activation by TAR RNA to allow for efficient synthesis of viral proteins as only Tat, PACT, and TAR RNA cannot block PKR activation efficiently in the absence of ADAR1 (Figure 6).

Several viruses have been shown to inactivate PACT function in the infected cells as PACT is involved both in activating PKR to suppress viral protein synthesis and in IFN production via RIG-I like receptors (RLRs) [88]. Middle East Respiratory Syndrome Coronavirus 4a protein [89], Herpes Simplex Virus US 11 protein [90,91], Ebola virus VP35 protein [92], Influenza virus NS1 protein [93] and orf virus ov20.2 protein [94] have been shown to inactivate PACT. Overall, our results show that the suppression of PACT activity to effectively inactivate PKR in HIV-1-producing cells is the result of the combined activity of the recruited ADAR1 that mediates PKR kinase inhibition, and Tat most probably stabilizes the complex formed by PKR, PACT and ADAR1. Any effect that Tat may have on PACT's function in RLR-mediated IFN production remains to be explored in future.

The results presented here shed light on how efficient translation of TAR-containing HIV-1-encoded RNAs takes place by suppressing PKR activation. The present work also presents us with new paradigms for testing possible ways to suppress HIV-1 viral protein synthesis. For example, if the formation of the inhibitory complex could be prevented by use of peptides that may block interaction between various components of this complex, we may be able to keep PKR activated in virally infected cells to prevent or at least partially block viral replication.

## Abbreviations

ADAR1: **a**denosine **d**eaminase **a**cting on **R**NA 1
CMV-TAR-LUC: CMV-TAR-luciferase
DMEM: Dulbecco's modified Eagle's medium
dsRNA: double-stranded RNA
DTT: dithiothreitol
IFN: interferon
HIV-1: human immunodeficiency virus type 1
ISG: interferon-stimulated gene
LTR: long terminal repeat
MEFs: murine embryonic fibroblasts
PACT: **p**rotein **act**ivator of PKR
PBMCs: peripheral blood mononuclear cells
PKR: protein kinase RNA-activated
PMSF: phenylmethylsulfonyl fluoride
RLR: RIG-I like receptor
TAR: trans-activation response element
TRBP: **T**AR **R**NA-**b**inding **p**rotein.
